# Three cases of diagnostic delay of type A acute aortic dissection

**DOI:** 10.1186/s43044-024-00444-y

**Published:** 2024-01-29

**Authors:** Takeshi Shimamoto, Sanae Tomotsuka, Makoto Takehara, Shinichi Tsumaru

**Affiliations:** Department of Cardiovascular Surgery, Hamamatsu Rosai Hospital, 25 Shogen-cho, Hamamatsu, Shizuoka 430-8525 Japan

**Keywords:** Type A aortic dissection, Diagnostic delay, Diagnostic error

## Abstract

**Background:**

Diagnostic delay (DD) can be lethal when patients with type A acute aortic dissection (TAAAD). We report 3 cases of DD associated with TAAAD.

**Case presentation:**

Case 1 is a female in her sixties presenting with severe back pain. A CT scan was taken, and TAAAD with a thrombosed false lumen was suspected by the radiology technician. He did not successfully transfer his concern to the physicians and the patient was sent home. The next day, she was transferred to another hospital with a recurrence of the symptom, and the diagnosis of TAAAD was made with a CT scan there. Case 2 was an 87-year-old female who was transferred to our hospital because of a loss of consciousness and bruises on the forehead. CT scan was taken and the displaced intimal flap in her aortic arch was overlooked by the part-time physician almost at the end of his shift. The diagnosis of TAAAD was made by the radiologist. Case 3 was the 44-year-old male who did not have health insurance and experienced severe back pain a few days before the visit to our clinic. On that day, he went to the nearby hospital’s emergency room, and only pain medication was prescribed. A few days later, a CT scan was taken at our hospital to investigate the cause of pyuria and the diagnosis of TAAAD was made.

**Conclusion:**

DD may be common and multifactorial in our practice. Physicians need to take every step to improve diagnostic accuracy.

## Background

Aortic dissections are associated with significant mortality and morbidity. They are caused by a tear in the intima of the aorta that extends into the media of the aortic wall. Blood flow through this tear leads to the formation of a false lumen bordered by the inner and outer layers of the media. Aortic dissections are classified by location and chronicity, with management strategies depending on the nature of the dissection. The Stanford method splits aortic dissections into type A and B, with type A dissections involving the ascending aorta [1].

Type A acute aortic dissection (TAAAD) is the most lethal vascular emergency. If left untreated, approximately 50% of patients die in the first 48 h. The mortality rate increases by 1–3% per hour [2]. Despite recent advances in diagnostic methods, misdiagnosis frequently occurs on initial evaluation in patients with symptoms mimicking those of acute myocardial infarction and other cardiovascular disorders [3]. However, with prompt diagnosis and treatment, the clinical outcome has been steadily improving, and the survival rate in 1 year has been reported as high as 90% [4]. Therefore, it is critical that the physician maintains a high clinical index of suspicion and utilizes a standardized algorithm to establish a prompt and accurate diagnosis of aortic dissection. We describe our experience of 3 cases of diagnostic delay (DD) with TAAAD.

## Case presentation

### Case 1

**Fig. 1:**
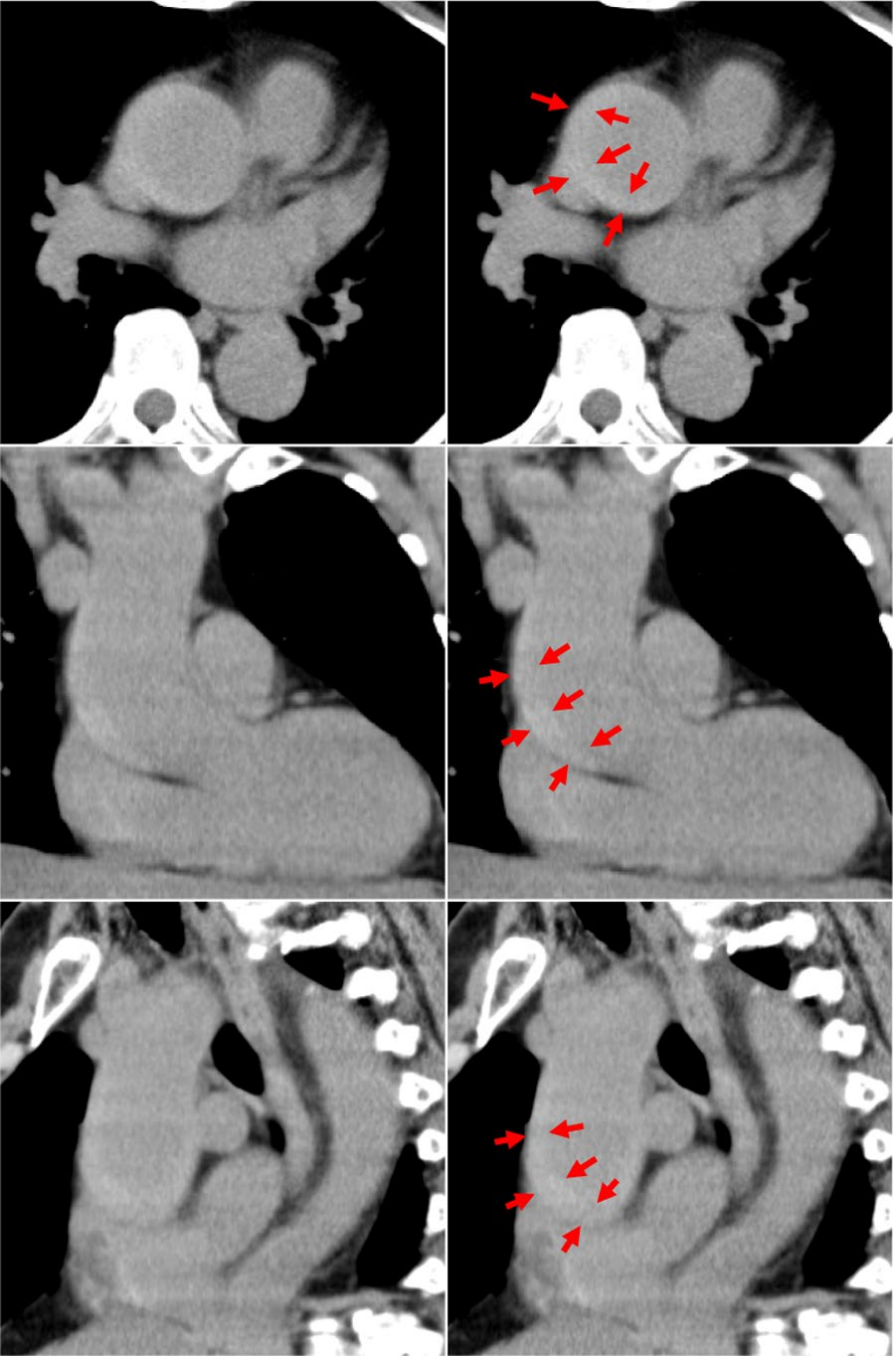
The axial, coronal, and sagittal views of the plain CT scan of case 1 from the top to the bottom. Note that difference in the contrast persisted throughout the ascending aorta (red arrows), indicating the presence of TAAAD with the thrombosed false lumen

At 7 pm one day, a female in her sixties was transferred to our hospital by ambulance because of the sudden onset of back pain. Her systolic blood pressure was over 150. The past history was unremarkable except for hypertension. The electrocardiogram showed no abnormality, and serum troponin was negative. Her white blood cell count was 15,600/µL, and her C reactive protein (CRP) was negative. Her renal function was normal with an estimated glomerular filtration rate of 61. Coagulation and fibrinolysis tests were ordered for ruling out aortic dissection and pulmonary embolism. However, the results were not available because of the sample shortage. On that day, the resident doctor was supposed to examine the patient first, and then report to the senior doctor who was not specialized in the cardiovascular field. Computed tomography (CT) scan without contrast material was taken (Fig. 1). In our hospital, the images were taken 5-mm axial view, 5-mm sagittal view, 3-mm coronal view by 64-row, and 0.5-mm multidetector CT (Aquilion 64, Canon Medical Systems Corporation, Tochigi, Japan). Although there was no immediate CT report interpreted by radiologists during the night shift in our hospital, the radiology technician suspected the presence of aortic dissection with a thrombosed false lumen in ascending aorta, and kindly conveyed his suspicion to the resident doctor, not the senior doctor, modestly with words that there was the “thickened wall in the ascending aorta,” hoping that the resident would realize this is potentially the sign of aortic dissection. The technician prepared coronal and sagittal images of the ascending aorta as well so that they could deny the artifact. The resident doctor and his senior doctor knew the disease of TAAAD. However, neither the resident nor the senior doctors knew the typical CT image of TAAAD of the thrombosed false lumen and ruled out the diagnosis of the dissection because the false lumen did not displace the true lumen, which was the typical sign of TAAAD with the patent false lumen. At 9 pm, 2 h after the onset of the symptom, the doctors permitted her to go home without any medication and asked her to see the cardiologist the next morning. At 6 am of the next morning, she was taken to the other hospital in the same city emergently with a recurrence of the symptom, and a CT scan there confirmed the diagnosis of TAAAD.

### Case 2

**Fig. 2:**
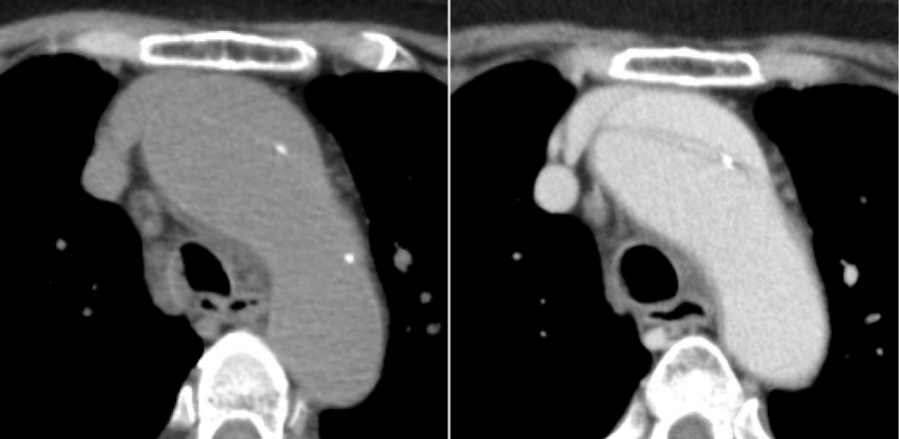
The axial view of the plain (left) and contrasted (right) CT scan of case 2. Note the presence of a displaced and slightly calcified intimal flap in the plain CT scan, which was confirmed with contrast

At 11 am of one day, an 87-year-old female was transferred to our hospital by ambulance with a bruise on the forehead. She told us that she was fallen on the floor of the bathroom for some hours. She was not able to tell us how long she was left on the floor, presumably because of her age or because of true syncope she suffered from the event. Her white blood cell count was elevated as high as 15,800/µL, and C reactive protein was 5.02 mg/dL. Her history was remarkable with multiple admission of aspiration pneumonia. The CT scan showed no cerebral bleeding, small pericardial effusion, dilated ascending aorta twice as large as descending aorta at the level of bronchial bifurcation, and mild bilateral pneumonia of the dorsal lower lobe. The physician in charge was a part-time doctor, and he was supposed to leave soon. He examined the images of the CT scan by himself and permitted her to go back to the nursing care house because the only abnormality he recognized was mild pneumonia and its degree was not severe enough for admission. While she prepared herself to leave the hospital, the radiologist noticed the slightly calcified and displaced intimal flap in the aortic arch in the CT scan and took the patient back for the CT scan with contrast material, which revealed the presence of TAAAD with a patent false lumen (Fig. 2). At 1 pm, she underwent emergency replacement of ascending aorta with the Dacron graft.

### Case 3

**Fig. 3:**
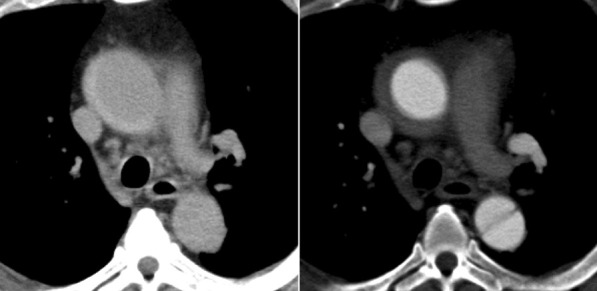
The axial view of the plain (left) and contrasted (right) CT scan of case 3. Note the difference in the contrast in the ascending aorta, which was confirmed with contrast

At 4 pm one day, a 44-year-old male with a body mass index of 45.7 experienced severe back pain a week before he visited our clinic. His history was remarkable with diabetes, hypertension, and dilatation of the ascending aorta as large as 4 cm. Soon after the onset of back pain, he visited the emergency room of a nearby general hospital, however, the in-charge physician only prescribed him analgesics. He did not order a CT scan presumably because he did not have insurance. The next day, he visited the nearby urologic clinic and found out that he had pyuria, proteinuria, elevated white blood cell count as high as 10,200/µL, and uncontrolled hypertension as high as 180/110. A few days later, because his back pain had not resolved, he was referred to our urology clinic with a tentative diagnosis of pyelonephritis refractory to antibiotics. The urologist in our clinic took a CT scan without contrast material, and our radiologist suspected the presence of TAAAD with the patent false lumen (Fig. 3). We confirmed his diagnosis of TAAAD with the CT scan with contrast material at 5 pm of the same day. We underwent semi-emergency total arch replacement.

## Discussion

Every disease is associated with misdiagnosis and diagnostic delay with significant variances of frequency and interval until reaching the correct diagnosis [5]. In the setting of the emergency department, aortic dissection is rare, and misdiagnosis and DD of aortic dissection are common, occurring in 14.1–39.4% of TAAAD patients [6] and are associated with worse outcomes [7]. In IRAD, DD was reported to be associated with a history of previous cardiac surgery, presentation without abrupt or any pain, and initial presentation to a nontertiary care hospital [8].

The diagnosis of TAAAD can be made by transthoracic or transesophageal echocardiography, CT, or magnetic resonance imaging (MRI). Although the transthoracic echocardiography is very useful in diagnosing aortic regurgitation, pericardial tamponade and cardiac wall motion abnormalities associated with coronary malperfusion caused by TAAAD, the value of transthoracic echocardiography is limited in patients with abnormal chest wall configuration, narrow intercostal spaces, obesity, pulmonary emphysema, and in patients on mechanical ventilation [9]. The transesophageal echocardiography is highly accurate for the detection of TAAAD; however it requires esophageal intubation, thus highly invasive. MRI is highly accurate imaging modality with a sensitivity of 95–98% and specificity of 94–98% for detecting aortic dissection [10]. However, its use in the emergency setting is very limited because of the limited access and cost. Moreover, it is time-consuming. Therefore, CT scan with contrast material is of paramount use because of relatively good access, acceptable cost, and good diagnostic accuracy. Although most of the diagnosis of aortic dissection was made based on the images of CT scan with contrast material, and the diagnosis was more likely to be missed or delayed when patients presented with atypical symptoms and physicians were not willing to infuse contrast material for potential renal damage and radiation.

The visualization of an intimal flap is the characteristic feature of TAAAD. If there is flow within both lumina, typical imaging features are probably present in the images of CT scan with contrast material. If the false lumen is thrombosed or there is no intimal tear to permit flow through the false lumen, a visible intimal flap may not be present. Secondary signs of TAAAD include an intramural or periaortic acute thrombus, which manifests as a high-attenuation cuff or crescent on the images of CT scan without contrast material as in our case 1. Other conditions that can reduce the diagnostic accuracy of the intimal flap include atypical configurations of the flap, such as seen with short dissections or with multiple false channels, in which the flaps are complex [11]. It is very important to pay attention to the small calcified masses, if any, located on the displaced flap, which renders physician accurate diagnosis of TAAAD with patent false lumen, even with CT scan without contrast material.

Every error in medical decision-making reportedly can be classified into three types: non-fault errors, system errors, and cognitive errors [12]. Non-fault errors include cases where the illness is silent, masked, or presented in such as an atypical fashion that divines the correct diagnosis. System errors reflect latent flaws in the health care system, including weak policies, poor coordination of care, inadequate training or supervision, defective communication, and the many system factors that detract from optimal working conditions such as stress, fatigue, and distraction. Cognitive errors are those in which the problem is inadequate knowledge faulty data gathering, inaccurate clinical reasoning, or faulty verification.

All the cases in this article were with diagnostic delay, not misdiagnosis. In our cases of diagnostic delay, every case has a component of non-fault error because the illness presentation is less severe compared with typical tearing pain of TAAAD or unstable hemodynamics. System error was more evident in case 1 where the radiology technician was almost confident about the presence of TAD, and poor communication between the radiology technician and doctors hampered the re-CT scan with contrast material or at least hospital admission for observation. The lack of knowledge of TAD with thrombosed false lumen is also attributable to system error as well. Case 3 was the case in which the definitive diagnostic step was delayed. According to the history the patient had given, the fact that he did not have health insurance might be a sufficient excuse for the physician not to take a CT scan during the emergency visit to the first hospital. Even in this circumstance, when the patient presented with severe back pain, the physician should have paid attention to the history of 4 cm ascending aorta dilatation since normal proximal ascending aorta was reported as 3.0 ± 0.4 cm [13].

Cognitive error was evident in case 2 in which the physician noticed the presence of aspiration pneumonia, but not the difference of contrast in the ascending aorta and size between ascending and descending aorta. This overlook might be influenced by the fact that the physician in charge was a part-time doctor and the timing of CT scan was the end of his working hour.

There might be several solutions to improve the diagnostic accuracy of TAAAD; careful medical examination including the characteristics of pain that patients experienced, improvement of communication within the medical team, timely and adequate education and training of TAAAD management, mutual assisting system between full-time and part-time physician, and so on to reduce diagnostic errors. The use of aortic dissection detection risk score [14] and echocardiography [15, 16] might be helpful to prevent misdiagnosis and DD. Either way, every single step should be taken to reduce diagnostic errors to save the lives of patients with TAAAD.

## Conclusions

TAAAD remains the life-threatening cardiovascular disorder facing physicians in the current era. A high clinical suspicion is crucial for accurate and prompt diagnosis. Aortic dissection is often misdiagnosed or diagnosed with significant delay, particularly when accompanied by less severe symptoms. A multidisciplinary and systematic approach with optimal teamwork and good communication is crucial to the diagnosis and treatment of TAAAD to improve the clinical outcome.

## Data Availability

The data underlying this article will be shared on reasonable request to the corresponding author.

## Abbreviations

TAAAD: Type A acute aortic dissection
DD: Diagnostic delay
CT: Computed tomography
MRI: Magnetic resonance imaging
